# Supplementary material for: Content-rich biological network constructed by mining PubMed abstracts
Source: BMC Bioinformatics. 2004 Oct 8;5:147. doi: 10.1186/1471-2105-5-147 (PMC528731; doi:10.1186/1471-2105-5-147)
Supplement: Additional File 2 — The original results of the above study (non-essential files are deleted to keep the file size under the limit set by BMC bioinformatics). [file 1471-2105-5-147-S2.bz2 › chilibotAdditionalFile2/dip05/582ID9571026E10950/html/GHR_GH1.html]

 


 **GHR** and **GH1** 
  
Found 60 abstracts in PubMed, retrieved 05.  
 

 What does Google say? 
 PDF only 
| .edu only 

---

- Hum Mutat, 2003   **Novel mutations of the growth hormone 1 **[GH1]** **GH1** gene disclosed by modulation of the clinical selection criteria for individuals with short stature.** .
  Subtle mutations in the growth hormone 1 **[GH1]** **GH1** gene have been regarded as a comparatively rare cause of short stature.
  Such lesions were sought in a group of 41 individuals selected for short stature, reduced height velocity, and bone age delay.
  a group of 11 individuals with short stature and idiopathic growth hormone deficiency IGHD .
  and a group of 154 controls.
  Heterozygous mutations were identified in all three groups but disproportionately in the individuals with short stature, both with odds ratio 25.2.
  95% CI, 5.1 132.2 and without odds ratio 3.6.
  95% CI, 1.0 12.9 IGHD.
  Twenty four novel **GH1** gene lesions were found.
  Thirteen novel missense mutations were characterized by assaying the signal transduction activity of in vitro expressed variants.
  six T27I, K41R, N47D, S71F, S108R, and T175A exhibited a reduced ability to activate the JAK STAT pathway.
  Molecular modeling suggested that both K41R and T175A might compromise GH receptor **[GHR]** binding.
  Seven GH variants R16C, K41R, S71F, E74K, Q91L, S108C, and a functional polymorphism, V110I manifested reduced secretion in rat pituitary cells after allowance had been made for the level of expression attributable to the associated **GH1** proximal promoter haplotype.
  A further leader peptide variant L 11P was not secreted.
  Eleven novel mutations in the **GH1** gene promoter were assessed by reporter gene assay but only two, including a GH2 gene templated gene conversion, were found to be associated with a significantly reduced level of expression.
  Finally, a novel intron 2 acceptor splice site mutation, detected in a family with autosomal dominant type II IGHD, was shown to lead to the skipping of exon 3 from the **GH1** transcript.
  A total of 15 novel **GH1** gene mutations were thus considered to be of probable phenotypic significance.
  Such lesions are more prevalent than previously recognized and although most may be insufficient on their own to account for the observed clinical phenotype, they are nevertheless likely to play a contributory role in the etiology of short stature.

  - Zhonghua Wai Ke Za Zhi, 2002    **Growth hormone insensitivity of rats under the endotoxemic condition** .
    OBJECTIVE To investigate the mechanism of growth hormone insensitivity of rats under the endotoxemic condition.
    METHODS Sprague Dawley rats n = 180 were injected endotoxin, TNF alpha, and IL 6 respectively.
    Part of endotoxin injected rats were treated with exogenous somatotropin **[GH1]** simultaneously, and all rats were killed at different time points.
    Liver expression of IGFI, **GHR** and SOCS 3 mRNA was detected by RT PCR, the levels of growth hormone GH were measured by radioimmunoassay, and the levels of TNF alpha and IL 6 were detected by ELISA.
    RESULTS Serum GH levels showed no significant change after endotoxin injection.
    liver IGFI and **GHR** mRNA expressions were obviously down regulated in endotoxemic rats, with the lowest decrease of 53% and 89% respectively.
    Athough SOCS 3 mRNA was weakly expressed in control rats, it was strongly up regulated in endotoxemic rats and the marked increase was 7.84 folds.
    The higher LPS dosage induced marked **GHR** mRNA down regulation and marked SOCS 3 mRNA up regulation.
    Exogenous GH made IGFI mRNA expression increase 25% in the control rats, but it did fail to prevent the decline in IGFI mRNA expression in endotoxemic rats.
    Endotoxin stimulated the production of TNF alpha and IL 6, and the elevated IL 6 levels showed a positive correlation with increased SOCS 3 mRNA expression.
    Liver **GHR** mRNA expression was obviously down regulated after TNF alpha iv injection, but for IL 6, it mainly up regulated the liver SOCS 3 mRNA expression.
    CONCLUSION The growth hormone insensitivity could be induced by LPS injection, which might be associated with down regulated **GHR** mRNA expression and up regulated SOCS 3 mRNA expression.
    The in vivo biological activities of LPS may be partialy mediated by TNF alpha and IL 6 at different aspects.

    - J Dairy Sci, 2002   **Effects of BST on oviductal and uterine genes encoding components of the IGF system in lactating dairy cows.** .
      Lactating Holstein cows, averaging 80 d in milk, were used to examine effects of exogenous bovine somatotropin **[GH1]** bST on oviductal and uterine genes encoding components of the insulin like growth factor IGF system.
      About 12 h before expected ovulation in an Ovsynch protocol, cows were assigned randomly to receive bST 500 mg.
      n = 11 or serve as untreated controls n = 10 .
      Cows that ovulated n = 9 bST, 8 control were divided within treatment to be sacrificed on d 3 or 7 postovulation.
      Samples of oviductal and intercaruncular endometrial tissue from oviducts and uterine horns ipsilateral to the corpus luteum CL were collected and immediately frozen at 80 degrees C for subsequent mRNA analyses.
      Northern blots revealed mRNAs for IGF II, IGF binding protein 2 IGFBP 2 , and IGFBP 3 in all oviductal and endometrial tissues.
      Significant amounts of IGF I and growth hormone receptor **[GHR]** 1A **GHR** 1A mRNAs were detected in uteri but not in oviducts.
      The bST treatment had no effect on amount of IGF I mRNA transcript in uterine endometrium.
      The mRNA encoding IGF II was induced by bST in oviducts collected on both d 3 and 7 but was down regulated in endometrium on d 7.
      Transcript of IGFBP 2 mRNA was greater in endometrial than oviductal tissues and did not differ between treatments.
      Both oviductal and endometrial IGFBP 3 mRNA concentrations increased between d 3 and 7 postovulation, with a tendency for greater endometrial IGFBP 3 mRNA in bST treated cows on d 7.
      On d 7, concentrations of endometrial **GHR** 1A mRNA were 30% lower in bST treated cows.
      Results indicate complex and tissue specific regulation of the uterine IGF system components by exogenous bST.
      Some of those biological responses to bST may be important in early development of bovine embryos.

      - Mol Cell Endocrinol, 2002   **Morphogenic and tumorigenic potentials of the mammary growth hormone growth hormone receptor **[GHR]** system.** .
        Due to the characteristics of the luteal phase of the ovarian cycle in the dog, which spans a prolonged time period, this species is a suitable model to study the role of progestins in both normal morphogenic and abnormal tumorigenic processes in the mammary gland.
        It has been convincingly shown that progestins, including endogenous progesterone, induce the synthesis of growth hormone GH in the normal and the tumorous canine mammary gland.
        The growth hormone receptor **[GHR]** **GHR** is also expressed in normal and tumorous canine mammary tissues and in this concise overview we highlight recent advances in our understanding of the significance of the GH **GHR** system for mammary gland patho biology.
        In an attempt to unravel the cellular and molecular mechanisms associated with the GH **GHR** system, we were able to show that both GH and **GHR** are differentially expressed in normal canine mammary tissues.
        Maximum expression of both GH and **GHR** occurs during the proliferation phase of the tissue, which links the progestin induced mammary GH synthesis to the progestin associated proliferation of epithelial cells in the mammary gland.
        Expression of the GH **GHR** system is also present in most canine mammary tumors, albeit that **GHR** expression may be downregulated in undifferentiated mammary carcinomas.
        Upon GH stimulation of the **GHR** positive CMT U335 canine mammary tumor cell line, the transcription factors STAT5A and STAT5B become phosphorylated on their tyrosine residues, which is likely to reflect the significance of mammary GH in vivo.
        Molecular analysis of the canine mammary **GHR** transcripts by RT PCR provided evidence for normal and alternative processing of the **GHR** primary transcript encoding the full length plasma membrane **GHR** and at least four putative GH binding proteins GHBPs , respectively.
        The translation products from the alternatively spliced **GHR** transcripts indicate an intact N terminal ligand binding domain and an unique C terminal portion, lacking the transmembrane domain and cytoplasmic tail.
        Thus, these proteins are considered to be able to bind GH, but have lost their signaling potential.
        The exact biological role of these GHBPs remains to be established, but GHBPs may have a transport function in the endocrine route, regulate the level of biologically available GH locally, or dominant negatively influence the full length plasmamembrane **GHR**.
        In dog mammary cancer specimens strongly reduced levels of alternatively spliced **GHR** transcripts were found compared to the non malignant mammary tissue.
        Notably, expression of both GH and **GHR** in mammary cancer cells is not restricted to dogs.
        Recent experiments generated evidence for GH and **GHR** expression in human breast cancer cells, and also in human prostate cancer cells, which represents another highly prevalent hormone sensitive human malignancy.
        In agreement with our findings in the dog, the expression of the hGH N **[GH1]** gene in human mammary cancer cells seemed to correlate positively with their progesterone receptor status, which warrants, in our opinion, a reconsideration of the role of progestins in breast cancer of women.
        In human prostate cancer cells four different hGH N **[GH1]** transcripts were detected, which encode classical 22 kDa GH and GH related proteins.
        Consistent with the findings on the canine **GHR**, different **GHR** transcripts in human mammary cancer cells and prostate cancer cells were detected encoding the full length plasmamembrane **GHR** and putative GHBPs.
